# Supplementary material for: Broccoli, Amaranth, and Red Beet Microgreen Juices: The Influence of Cold-Pressing on the Phytochemical Composition and the Antioxidant and Sensory Properties
Source: Foods. 2024 Feb 29;13(5):757. doi: 10.3390/foods13050757 (PMC10930722; doi:10.3390/foods13050757)
Supplement: Supplementary file 1 [file foods-13-00757-s001.zip › foods-2865187-supplementary.pdf]

foods

MDPI

Article

Broccoli, Amaranth, and Red Beet Microgreen Juices: The Influence of Cold-Pressing on the Phytochemical Composition and the Antioxidant and Sensory Properties

Spasoje D. Belošević [1,†], Danijel D. Milinčić [2,†], Uroš M. Gašić [3], Aleksandar Ž. Kostić [2], Ana S. Salević-Jelić [1], Jovana M. Marković [1], Verica B. Đorđević [4], Steva M. Lević [1], Mirjana B. Pešić [2,*] and Viktor A. Nedović [1,*]

[1] Food Biotechnology Laboratory, Department of Food Technology and Biochemistry, Faculty of Agriculture, University of Belgrade, Nemanjina 6, 11080 Belgrade, Serbia; sbelosevic@agrif.bg.ac.rs (S.D.B.); ana.salevic@agrif.bg.ac.rs (A.S.S.-J.); jovana.markovic@agrif.bg.ac.rs (J.M.M.); slevic@agrif.bg.ac.rs (S.M.L.)
[2] Food Chemistry and Biochemistry Laboratory, Department of Food Technology and Biochemistry, Faculty of Agriculture, University of Belgrade, Nemanjina 6, 11080 Belgrade, Serbia; danijel.milincic@agrif.bg.ac.rs (D.D.M.); akostic@agrif.bg.ac.rs (A.Ž.K.)
[3] Department of Plant Physiology, Institute for Biological Research Siniša Stanković-National Institute of Serbia, University of Belgrade, Bulevar Despota Stefana 142, 11060 Belgrade, Serbia; uros.gasic@ibiss.bg.ac.rs
[4] Department of Chemical Engineering, Faculty of Technology and Metallurgy, University of Belgrade, Karnegijeva 4, 11000 Belgrade, Serbia; vmanojlovic@tmf.bg.ac.rs
* Correspondence: mpesic@agrif.bg.ac.rs (M.B.P.); vnedovic@agrif.bg.ac.rs (V.A.N.); Tel.: +381-11-441-3315 (M.B.P.); +381-441-3154 (V.A.N.)
† These authors equally contributed to this work.

Table S1. Equation parameters and correlation coefficient (R^2) of the phenolic standards used for quantification.

Standards	y=a*x±b	R^2
Sinapic acid	y= 83605.5527 * x - 8503.3101	0.9920
Apigenin	y= 215501.5584 * x + 64892.2142	0.9942
